# FACTORS INFLUENCING STAFF-RESIDENT INTERACTIONS IN NURSING HOMES

**DOI:** 10.1093/geroni/igz038.1860

**Published:** 2019-11-08

**Authors:** Anju Paudel, Elizabeth Galik, Barbara Resnick

**Affiliations:** 1 University of Maryland School of Nursing, Maryland, United States; 2 University of Maryland School of Nursing, Baltimore, Maryland, United States

## Abstract

Staff-resident interaction is an integral part of daily life of nursing home residents and has an influence on residents’ well-being. However, less is known about the factors that influence these interactions. The purpose of this study was to describe the quality of interaction between staff and residents with dementia in nursing homes, and explore the factors associated with ‘positive’ and ‘negative/neutral’ interactions. This cross-sectional analysis utilized baseline data from the first two cohorts in a randomized clinical trial, EIT-4-BPSD, including 338 residents from 35 nursing homes. Generalized linear mixed model (GLMM) was used to explore the factors associated with interactions. It was hypothesized that the resident factors (age, gender, race, marital status, cognition, comorbidities, depressive symptoms, agitation, functional status) and facility factors (facility ownership, facility size, RN hours, LPN hours, CNA hours, and star rating) would be associated with staff-resident interactions. The staff-resident interactions were mostly positive. Overall, the models for ‘positive interactions’ and ‘negative/neutral interactions’ correctly classified 82.8% and 85.3% of the cases respectively. Both ‘positive’ and ‘negative/neutral’ interactions were significantly associated with marital status, and profit status of the facility. Being married and living in a not for profit facility was associated with lower odds of positive interaction and higher odds of negative/neutral interaction. There is some evidence that marital status influences staff-resident interactions and that profit status of facilities are associated with staff resident interactions. Future studies could explore staff factors such as consistent assignment, job satisfaction, staff characteristics, and training.

